# B Cells in Tumor Microenvironment Associated With The Clinical Benefit to Programmed Cell Death Protein-1 Blockade Therapy in Patients With Advanced Esophageal Squamous Cell Carcinoma

**DOI:** 10.3389/fonc.2022.879398

**Published:** 2022-06-29

**Authors:** Jhe-Cyuan Guo, Chia-Lang Hsu, Yen-Lin Huang, Chia-Chi Lin, Ta-Chen Huang, I-Chen Wu, Chen-Yuan Lin, Ming-Yu Lien, Hung-Yang Kuo, Ann-Lii Cheng, Chih-Hung Hsu

**Affiliations:** ^1^ Department of Medical Oncology, National Taiwan University Cancer Center, Taipei, Taiwan; ^2^ Department of Oncology, National Taiwan University Hospital, Taipei, Taiwan; ^3^ Graduate Institute of Clinical Medicine, National Taiwan University College of Medicine, Taipei, Taiwan; ^4^ Department of Medical Research, National Taiwan University Hospital, Taipei, Taiwan; ^5^ Graduate Institute of Oncology, National Taiwan University College of Medicine, Taipei, Taiwan; ^6^ Department of Pathology, National Taiwan University Cancer Center, Taipei, Taiwan; ^7^ Division of Gastroenterology, Department of Internal Medicine, Kaohsiung Medical University Hospital, Kaohsiung, Taiwan; ^8^ Division of Hematology and Oncology, Department of Internal Medicine, China Medical University Hospital, Taichung, Taiwan

**Keywords:** esophageal squamous cell carcinoma, B cell, immune checkpoint inhibitor, prognosis, efficacy

## Abstract

**Background:**

B cells and B cell-related gene signatures in the tumor microenvironment (TME) are associated with the efficacy of anti-programmed cell death-1 (anti-PD-1) therapy in several cancer types, but not known for esophageal squamous cell carcinoma (ESCC).

**Patients and Methods:**

Patients with advanced ESCC receiving anti-PD-1/PD-L1-based therapy were retrospectively included. A targeted RNA profiling of 770 immune-related genes from archival ESCC tissues was performed. Differential immune-related pathways and the levels of infiltrating immune cells were estimated through Gene Set Enrichment Analysis and CIBERSORT, respectively. CD19 and CD138 expression were evaluated through immunohistochemistry (IHC). The markers evaluated were correlated with clinical benefit (CB; defined as either objective response or stable disease for ≥6 months) and survival.

**Results:**

A total of 64 patients were enrolled. The transcriptome analysis based on 25 patients revealed that B cell signature was significantly increased in patients with CB (*P* <.05) and correlated with a longer PFS (*P* = .032) and OS (*P* = .013). Multiple genes representative of B cells, B cell functions, and plasma cells were upregulated in patients with CB. On further analysis of B cell subtypes in patients with CB, increase of naïve B cells (*P* = .057) and plasma cells (*P* <.01) was found but not memory B cells (*P* = .27). The CD19 expression in tumor stroma, detected by IHC, was higher in patients with CB (*P* = .033).

**Conclusion:**

B cells in the TME were associated with CB in patients with advanced ESCC receiving anti-PD-1/PD-L1-based therapy.

## Introduction

In 2018, esophageal cancer (EC) ranked as the 7^th^ most commonly diagnosed and the 6^th^ most lethal cancer type worldwide (1). Esophageal squamous cell carcinoma (ESCC), which is the major type of EC in Asia and Africa, comprises the vast majority of EC worldwide. ESCC differs from esophageal adenocarcinoma with regard to geographic distribution, risk factors, and genetic alterations (2).

Immune checkpoint inhibitors (ICIs), particularly anti-programmed cell death protein-1 (anti-PD-1) and anti-PD ligand-1 (anti-PD-L1) therapy have changed the treatment landscape of many cancer types, including EC (3). Anti-PD-1 therapy has outperformed chemotherapy as the second-line systemic therapy for patients with recurrent or metastatic ESCC in multiple phase III trials (4–6). However, the response rate to anti-PD-1 monotherapy is around 15~ 20% in patients with advanced ESCC. Potential biomarkers with prognostic significance or predicting efficacy of anti-PD-1 ICIs for ESCC, such as tumor mutational burden, T-receptor clonality and molecular tumor burden index from peripheral blood, and tumor-infiltrating lymphocytes (TILs), are under active investigation (7–10).

B cells and plasma cells located at tumors and tumor-draining lymph nodes could shape the antitumor response. In a tumor-associated tertiary lymphoid structure (TLS), B cells and T cells together enhance antitumor immunity (11). Contrarily, several studies have suggested that tumor-infiltrating B cells and intratumorally produced antibodies may exhibit protumor effects (11). Several early studies exploring the role of B cells in the efficacy of ICIs yielded inconsistent results (12–15). Recently, 3 comprehensive translational studies have demonstrated the association of B cells or TLS with the efficacy of ICIs in melanoma and soft tissue sarcoma (16–18). Whether this association also exists in ESCC is unknown.

In this retrospective study, we enrolled patients with advanced ESCC treated with ICIs and investigated the association of B cells in ESCC tumor tissues with the clinical benefit (CB) to anti-PD-1 therapy. Our data, based on transcriptome analysis and immunohistochemistry (IHC) of B cells in ESCC tumor tissues, suggest an association of B cells in the tumor microenvironment (TME) with improved efficacy of anti-PD-1 therapy in patients with ESCC.

## Materials and Methods

### Patients

Patients with recurrent or metastatic ESCC who underwent anti-PD-1 or anti-PD-L1 therapy or an anti-PD-1/PD-L1-based immunotherapy combination between August 1, 2015, and February 28, 2020, were retrospectively identified from 3 medical centers in Taiwan. Patients with formalin-fixed paraffin-embedded (FFPE) archival tumor tissue obtained before ICI administration were enrolled. A variety of tissue sources including primary site with or without neoadjuvant therapy and recurrent or metastatic sites were used. Because the majority of available archival tissues is from biopsy, many of the archival tissues from a biopsy are not adequate enough for the gene expression analysis. Anti-PD-1 and anti-PD-L1 therapy were administered every 2 to 4 weeks on the basis of the doses and schedules recommended for each individual agent. The response to the therapy was evaluated every 8 to 12 weeks through image studies by using RECIST 1.1. This study was approved by Research Ethics Committee of National Taiwan University Hospital (approval No.: 201612155RINB).

### Gene Expression Analysis

Bulk RNA was extracted from FFPE archival tumor tissues by using the Qiagen’s miRNeasy FFPE Kit (Qiagen, Valencia, CA). The quality of extracted RNA was analyzed using Bioanalyzer (model 2100, Agilent Technologies, Santa Clara, CA, USA). Gene expression analysis was conducted on the NanoString nCounter platform with the Human PanCancer Immune Profiling panel (NanoString Technologies, Seattle, WA, USA). The gene expression was processed through the conversion of raw counts into counts per million followed by normalization with a trimmed mean of M-values (19). The abundance of tumor-infiltrating immune cells was estimated through the mean of the log_2_-transformed normalized expression levels of the genes specified for individual immune cell types (20). An alternative method, CIBERSORT with the LM22 signature matrix, was employed to profile tumor-infiltrating immune cells (21). Differentially expressed genes between tumors with and without CB were assessed using limma (22). Differential activation of immune-related pathways was evaluated using Gene Set Enrichment Analysis (GSEA) (23). The gene sets used in GSEA was obtained from Molecular Signatures Database v7.0 (MSigDB) (24). Gene expression profiling data of patients with ESCC from The Cancer Genome Atlas (TCGA) dataset were retrieved for evaluating the prognostic effect of different tumor-infiltrating immune cells.

### IHC Staining

CD19 expression (BT51E; 1:100 dilution; Leica; B cell) and CD138 (ZM249; ready to use; ZETA; plasma cell) was determined through IHC staining. In brief, the FFPE tissue section was deparaffinized and then rehydrated. Thereafter, cell conditioning was used for antigen unmasking. The primary antibody, secondary antibody, and 3, 3’-diaminobenzidine detection were applied in accordance with the protocol from manufacturer instructions. Finally, hematoxylin was applied for counterstaining. The CD19 and CD138-expressing cells were semiquantitatively assessed (by co-author Huang Y-L, a pathologist focusing on thoracic pathology) using a method previously described for scoring TILs in solid tumors, and were scored for those in the intratumoral and stromal compartments, respectively (25).

### Statistical Analyses

The data cutoff date was August 31, 2020. As a surrogate for the efficacy of anti-PD-1 therapy, CB was defined as complete response, partial response, or stable disease for ≥6 months according to the best tumor response per RECIST 1.1. Gene expression analysis was conducted and values plotted using R (v. 3.5.1; http://www.r-project.org/). Progression-free survival (PFS) was defined as the time between the date of starting therapy and the date of progressive disease, death, or the final follow-up (censored). Overall survival (OS) was defined as the time between the date of starting therapy and the date of death or final follow-up (censored). The Mann–Whitney test was used to compare the IHC staining between tumors with and without CB by GraphPad Prism. The Cox proportional hazards model using the “coxph” function from the R “survival” package was employed. Kaplan–Meier survival curves and scatter plots were plotted using GraphPad Prism version 5.01 (GraphPad Software, San Diego, CA, USA).

## Results

### Patient Characteristics and Clinical Outcomes

**Figure 1:**
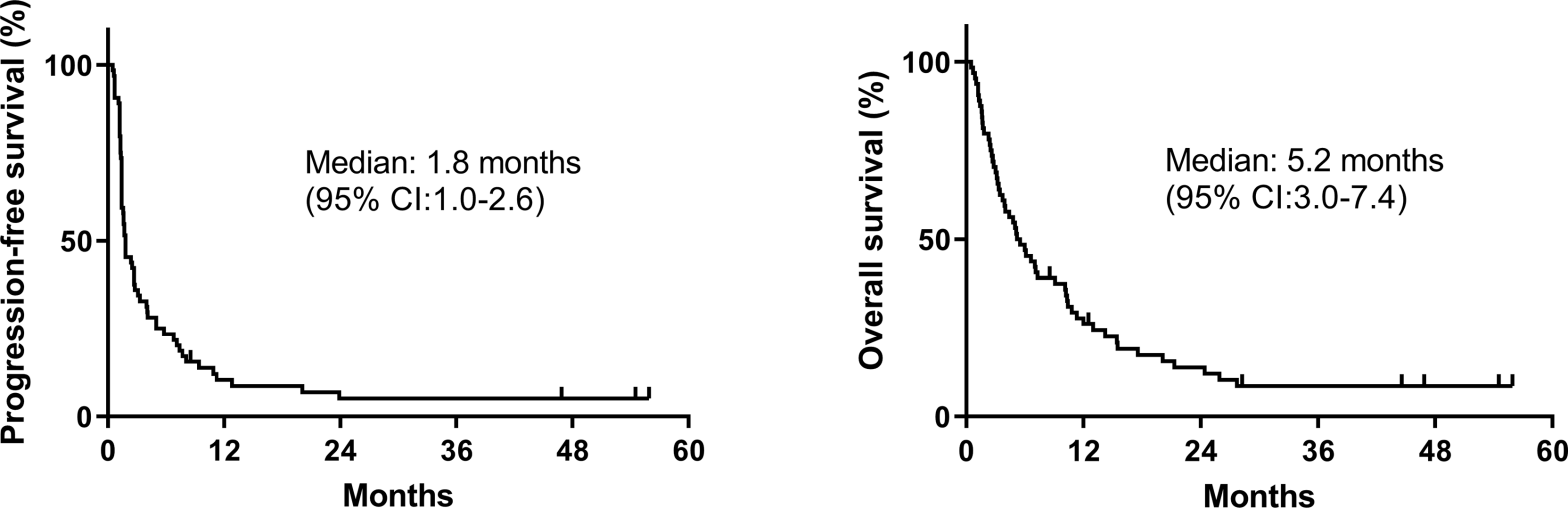
Kaplan–Meier survival curves of progression-free survival and overall survival of the entire cohort.

In total, 64 patients were enrolled. Of these, 38 received anti-PD-1 or anti-PD-L1 therapy, whereas 26 were treated with an anti-PD-1/PD-L1-based immunotherapy combination. The majority of patients were male and had good performance status. More than 60% patients had their disease involving at least 2 organ sites; more than one-third of patients had been treated with 2 lines of systemic therapy for advanced ESCC. In the 25 patients whose tumors were subjected to gene expression analysis (the gene expression cohort), higher proportion of patients were recurrent disease and had received esophagectomy compared with total patient cohort (IHC cohort) (*P* = .016 and.005, respectively). The patients’ pertinent characteristics are summarized in **Table 1**. Overall, the response rate was 17%, and the CB rate was 25%. The median PFS and OS were 1.8 (95% CI: 1.0-2.6) and 5.2 (95% CI: 3.0-7.4) months, respectively (**Figure 1**).

**Table 1:** Baseline characteristics and response to therapy.

Characteristic	Total cohort (N = 64) (%)	GE cohort (N = 25) (%)	P-value ^α^(GE vs Total)
Median age, years (range)	59.0 (37.2-82.8)	59.6 (45.0-77.8)	0.812
ECOG performance status 0-1 >1	55 (86) 9 (14)	24 (96) 1 (4)	0.271
Sex Male Female	63 (98) 1 (2)	25 (100) 0 (0)	1.000
Primary esophageal cancer Cervical and upper thoracic Middle thoracic Lower thoracic	18 (28) 20 (31) 26 (41)	4 (16) 9 (36) 12 (48)	0.491
Differentiation Well Moderate Poor Unknown	2 (3) 31 (48) 15 (23) 16 (25)	1 (4) 15 (60) 5 (20) 4 (16)	0.741
Disease status Recurrent De novo metastatic	43 (67) 21 (33)	23 (92) 2 (8)	0.016
Esophagectomy Yes No	27 (42) 37 (58)	19 (76) 6 (24)	0.005
Tumor burden - involved sites 1 >1	24 (38) 40 (63)	10 (40) 15 (60)	0.827
Treatment^β^ Anti-PD-1/PD-L1 alone Anti-PD-1/PD-L1-based combination	38 (59) 26 (41)	11 (44) 14 (56)	0.190
Prior lines of systemic therapy 0-1 ≥2	40 (63) 24 (38)	16 (64) 9 (36)	0.895
Best response Complete response Partial response Stable disease Progressive disease Not evaluable	0 (0) 11 (17) 15 (23) 28 (44) 10 (16)	0 (0) 5 (20) 7 (28) 12 (48) 1 (4)	0.519
Clinical benefitγ Yes No	16 (25) 48 (75)	9 (36) 16 (64)	0.299

^αγ^Mann-Whitney U test for age and Chi-square test for other characteristics. ^β^Anti-PD-1/PD-L1 alone includes nivolumab, pembrolizumab, tislelizumab, spartalizumab, and durvalumab. Anti-PD-1/PD-L1-based combination includes durvalumab plus tremelimumab, nivolumab plus ipilimumab, spartalizumab plus NIS793, and bintrafusp alfa. ^γ^Clinical benefit: complete response, partial response and stable disease ≥ 6 months. ECOG, Eastern Cooperative Oncology Group; GE, gene expression; PD-1/PD-L1, programmed cell death protein-1/PD ligand 1.

### Expression of Immune-Related Genes in ESCC Tumors From Patients With and Without CB

**Figure 2:**
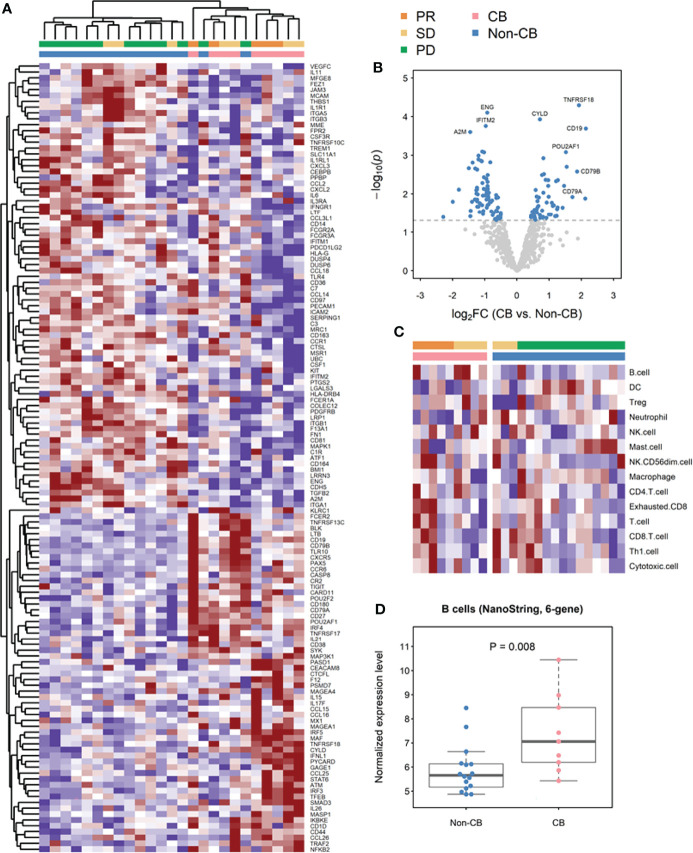
Transcriptional analyses of formalin-fixed paraffin-embedded (FFPE) archival tumor specimens from patients with advanced esophageal squamous cell carcinoma (ESCC) treated with anti-PD-1 or anti-PD-L1-based immunotherapy. **(A)** Unsupervised hierarchical clustering of differentially expressed genes (DEGs) by using NanoString platform analysis. **(B)** Volcano plot depiction of DEGs based on clinical benefit (CB). **(C)** Supervised clustering of ESCC FFPE archival tumor specimens based on CB (n = 16 non-CB and 9 CB), displaying nCounter scores. **(D)** Log_2_-transformed normalized expression levels of B cells based on nCounter scores clustered by CB. (DC, dendritic cells; NK cells, natural killer cells; PR, partial response; SD, stable disease; PD, progressive disease).

In total, 25 FFPE archival tissues (9 CB and 16 non-CB) were subjected to gene expression analysis on the basis of 770 immune-related genes included in the Human PanCancer Immune Profiling panel (NanoString). An unsupervised hierarchical clustering of differentially expressed genes is illustrated in **Figure 2A**, which revealed that a substantial number of genes were indeed differentially enriched in patients with and without CB. The differentially expressed genes that were significantly increased in patients with CB versus those without CB are further shown in **Figure 2B** (top right) through a volcano plot. Among them, *CD19*, *CD79A*, *CD79B*, *PU2AF1*, and *CYLD* genes are related to B cells and B cell functions.

### Gene Expression Signatures of B Cells and B Cell Subpopulations in ESCC Tumors Correlated With CB

**Figure 3:**
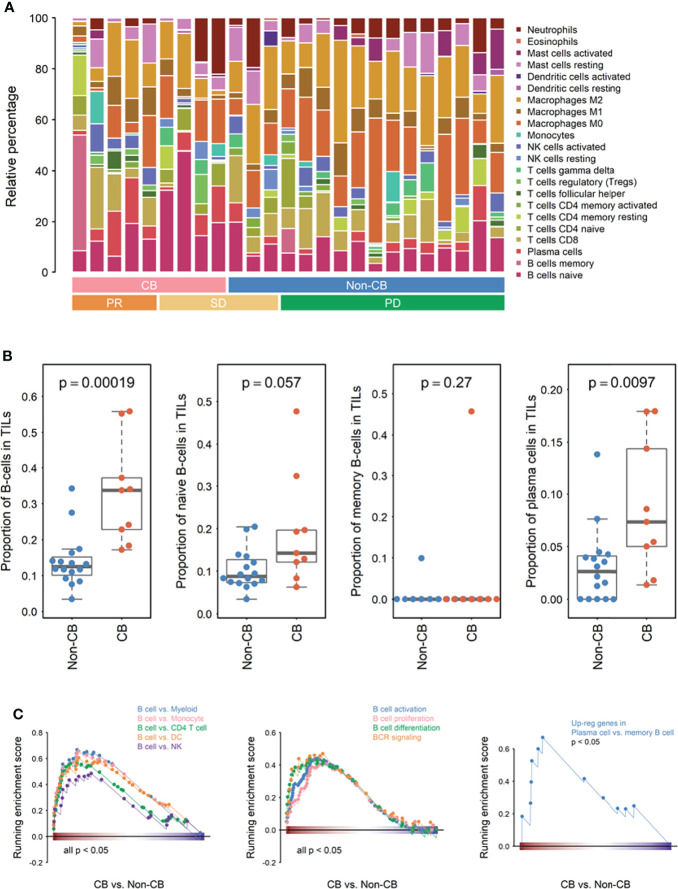
Transcriptional analyses of immune cell subpopulations classified using CIBERSORT and differential immune-related pathways evaluated using Gene Set Enrichment Analysis (GSEA). **(A)** Relative percentage of immune cell subpopulations clustered based on clinical benefit (CB) (n = 16 non-CB and 9 CB) and response (PR, partial response; SD, stable disease; and PD, progressive disease). **(B)** Scatter plots demonstrating the percentage of B cells and B cell subpopulations as indicated between the CB (n = 9) and non-CB (n = 16) groups. **(C)** GSEA plots of immune cells, B cell function, and plasma cells compared with memory-B-cell-related genes expressed in CB (n = 9) and non-CB (n = 16) groups.

To evaluate whether the abundance of B cells in the TME was indeed increased in patients with CB compared with those without CB, we first analyzed the data by using the immune cell scores previously defined by the NanoString nCounter platform with the Human PanCancer Immune Profiling panel (20). B cells was the most highly differentially expressed cell types between CB and non-CB groups of patients, and the B-cell score was significantly higher in the CB group than in the non-CB group (*P* = .008; **Figures 2C, D**). B-cell score also trended to be higher in the CB group than the non-CB group even after considering the archival tissue sources and prior treatment status (**Supplementary Material** and **Supplementary Figure 1**). To confirm this observation and further explore the specific B cell subpopulations associated with CB, we used an alternative immune cell profiling method: CIBERSORT with the LM22 signature matrix (**Figure 3A**). The CIBERSORT method confirmed that the total B cell population in tumor infiltrating leukocytes was significantly increased in ESCC tumors of patients with CB compared with those without CB (*P* <.01). Among the B cell subpopulations evaluated, naïve B cells tended to be more abundant in patients with CB (*P* = .057), and plasma cells were significantly increased in patients with CB (*P* <.01); conversely, no significant difference was discovered in memory B cells between patients with and without CB (*P* = .27; **Figure 3B**).

GSEA was performed to identify the differential immune-related pathways and immune cell-specific expressed genes obtained from MSigDB. We first found that gene sets which are defined as high expression in B cells compared with other immune cells, including myeloid cells, monocyte, CD4 T cells, dendritic cells, and NK cells were consistently upregulated in patients with CB (*P* <.05 for all comparisons; **Figure 3C**). Additionally, various B cell functions, including activation, proliferation, differentiation, and B cell receptor signaling, were upregulated in patients with CB (*P* <.05 for all comparisons; **Figure 3C**). Moreover, the level of plasma cell-related genes compared with memory B-cell-related genes was significantly upregulated in patients with CB (*P* <.05; **Figure 3C**), a finding that is consistent with our analysis conducted using the CIBERSORT method (**Figure 3B**).

### B Cell Gene Signature in ESCC Tumors Correlated With Improved Survival in Patients Treated With Anti-PD-1 ICIs

**Figure 4:**
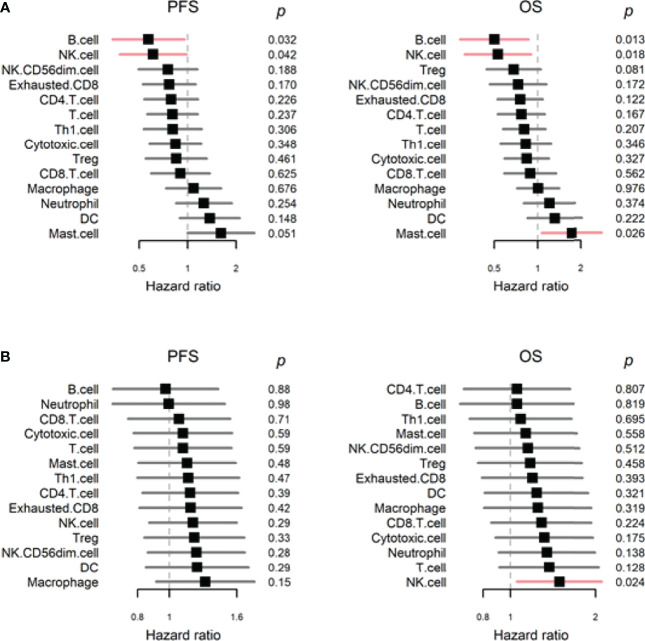
Progression-free survival (PFS) and overall survival (OS) analyses of proportional values for different immune cell signatures in **(A)** our cohort and **(B)** The Cancer Genome Atlas cohort. (DC, dendritic cells; NK cells, natural killer cells).

We further analyzed the prognostic significance of different immune cell signatures in our cohort of patients with ESCC by using the Cox proportional hazards model. As summarized in **Figure 4A**, high B cell and NK cell signatures were associated with longer PFS (*P* = .032, hazard ratio [HR] = 0.57 [95% CI: 0.34-0.95] and *P* = .042, HR = 0.61 [95% CI: 0.38-0.98], respectively) and OS (*P* = .013, HR = 0.50 [95% CI: 0.29-0.86] and *P* = .018, HR = 0.53 [95% CI: 0.31-0.90], respectively), whereas high mast cell signature was associated with reduced OS (*P* = .026, HR = 1.72 [95% CI: 1.07-2.78]). To understand whether these immune cell signatures have similar prognostic significance in patients with ESCC with localized disease, we analyzed the gene expression data of patients with ESCC from the TCGA dataset. In the TCGA dataset, all patients with ESCC received surgical intervention for localized disease, and were enrolled in the era when anti-PD-1 ICIs were unavailable for human cancer treatment (26). Our analysis of the TCGA dataset showed that the B cell signature was not associated with PFS or OS, and high NK cell signature was associated with reduced OS (*P* = .024, HR = 1.49 [95% CI: 1.05-2.10]; **Figure 4B**).

### IHC-Detected CD19 and CD138-Expressing Cells in ESCC Tumor Tissues From Patients With and Without CB

**Figure 5:**
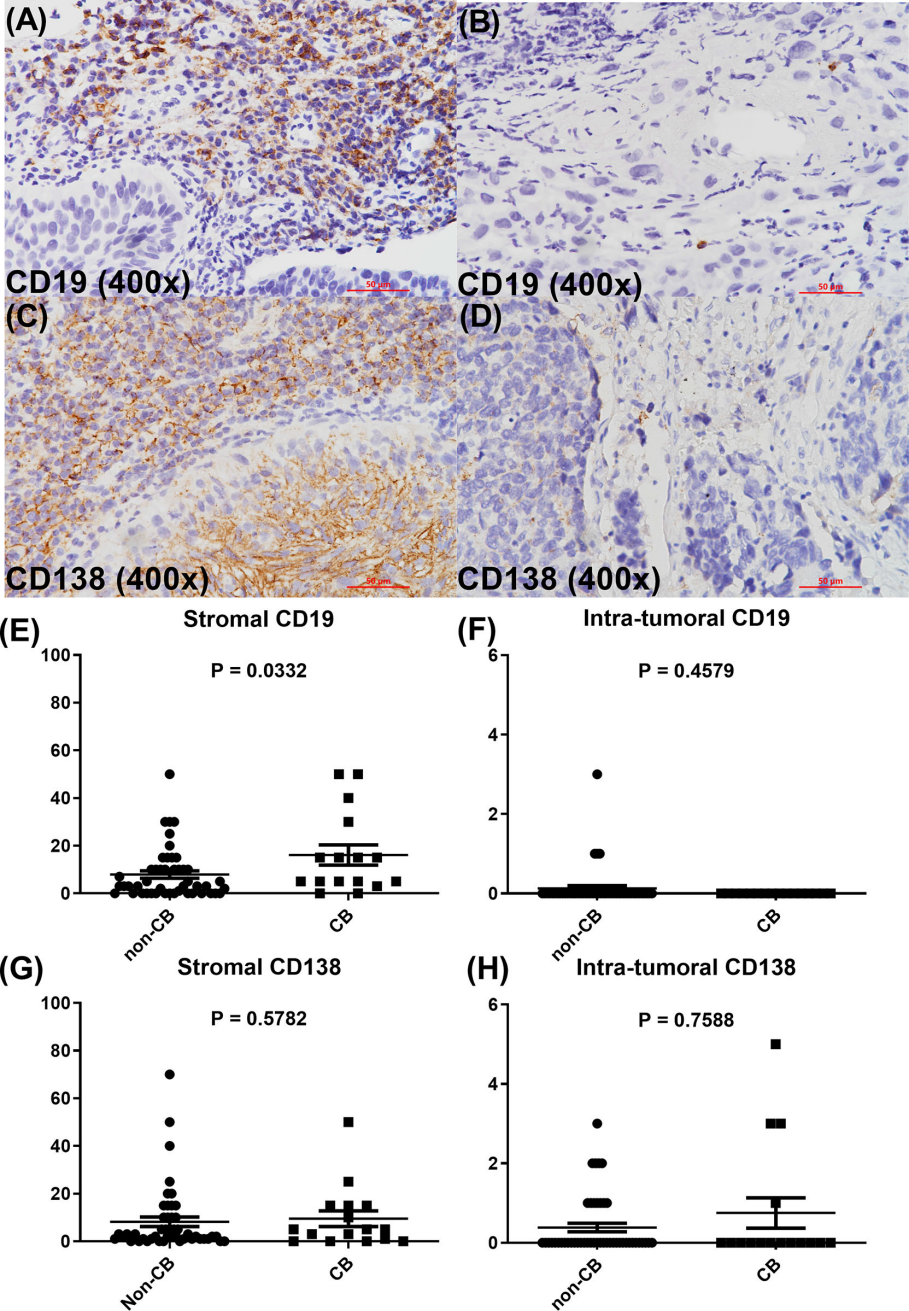
Representative figures of stromal and intratumoral CD19 **(A, B)** and CD138 expression **(C, D)**. Scatter plots of the immunohistochemistry CD19 **(E, F)** and CD138 **(G, H)** expression levels as indicated between CB and non-CB groups.

To further confirm the findings of gene expression analysis, IHC studies of CD19 and CD138 expression were conducted in tumor tissues of 64 patients (16 CB and 48 non-CB). Representative pictures of stromal and intratumoral CD19 (**Figures 5A, B**) and CD138 (**Figures 5C, D**) were demonstrated. We found that CD19 expression in stroma was significantly increased in patients with CB compared with those without CB (*P* = .033; **Figure 5E**). CD19 expression in stroma also trended to be increased in patients with CB compared with those without CB even after considering the archival tissue sources and prior treatment status (**Supplementary Material** and **Supplementary Figure 1**). CD19 expression in the intratumoral compartment was very low and not significantly different between patients with and without CB (**Figure 5F**). CD138 expression in stroma and intratumoral compartment was not significantly different between patients with and without CB (**Figures 5G, H**).

## Discussion

Immunotherapy, particularly ICI therapy, has changed the landscape of therapy for ESCC (4–6). However, only some patients with ESCC benefit from ICI treatment. This retrospective study revealed that B cells in the TME, assessed through gene expression profiles and IHC, were associated with CB and favorable prognosis in patients with advanced ESCC treated with anti-PD-1-based or anti-PD-L1-based immunotherapy.

In the current study, we found that a strong B cell signature, defined by nCounter Human PanCancer Immune Profiling or CIBERSORT, was associated with CB in patients with ESCC treated with anti-PD-1-based or anti-D-L1-based immunotherapy. Our observation is consistent with those of other studies involving patients with melanoma, sarcoma, and non-small cell lung cancer (NSCLC) (13, 15–18, 27). Nevertheless, B cell subpopulations associated with the efficacy of ICI treatment vary among these studies including ours. Griss et al. found that advanced melanoma responding to ICIs had higher naïve-like and plasmablast-like B cell frequencies in pretherapy tumor samples (13). Helmink et al. found that melanoma tumors in responders had high frequencies of switched memory B cells and plasma cells, whereas nonresponders had high frequencies of naïve B cells (17). Patil et al. found that B cells and plasma cells are present in TLS in NSCLC tumors and there is a strong association of increased intratumoral plasma cells with longer OS in patients with advanced NSCLC treated with anti-PD-L1 therapy (27). In our study, plasma cell gene signature correlated well with CB, but plasma cell detected by IHC-expression of CD138 was not significantly associated with CB. The discrepant results from two different assays may be contributed by the relatively small sample size of our study, the imbalanced patients’ characteristics of the two studied cohorts, and the intratumor heterogeneity in immune infiltrates. Additional studies are necessary to solve this discrepancy.

In our gene expression analysis study, we found a significantly higher frequency of plasma cells and a trend of increased frequency of naïve B cells in ESCC tumors from patients with CB; we did not find a significant difference in memory B cells between patients with and without CB. Our additional analysis showed that upregulation of B cell function-related genes—including activation, proliferation, differentiation, and B cell receptor signaling—in ESCC tumors from the CB group. These data, taken together, support the notion that B and T cells in combination could modify adaptive immunity against tumors, and the upregulation of B cell function may in some way contribute to the antitumor efficacy of anti-PD-1-based treatment.

The prognostic effect of B cells has not yet been comprehensively investigated in ESCC. Ma et al. found that high infiltration of immune cells (T cells, B cells, and macrophages) in tumor tissues of ESCC was associated with favorable OS in patients with localized ESCC treated with surgery (28). In the current study focusing on patients with advanced ESCC, we demonstrated that strong B cell and NK cell signatures were associated with longer PFS and OS, whereas a high mast cell signature was associated with reduced OS. In the TCGA data cohort based on patients with early stage localized ESCC, no prognostic significance was observed for the B cell signature, and a negative prognostic effect was observed for the NK cell signature, a finding contradictory to aforementioned findings from our patient cohort with advanced ESCC. These seemingly inconsistent observations suggest that the B cell signature may be a potential predictive biomarker associated with the efficacy of anti-PD-1-based treatment in patients with advanced ESCC because it does not exhibit prognostic significance in patients with ESCC of earlier stages not receiving ICI treatment. Furthermore, the contradictory prognostic significance of the NK cell signature between our advanced ESCC cohort and TCGA cohort are in line with several previous studies which also reported inconsistent results regarding the prognostic effects of NK and other cells in ESCC (29–31). Additional studies are warranted to investigate the prognostic significance of different immune cells in ESCC.

This study has several limitations. First, the sample was small, consisting of only 64 patients with ESCC. Only 25 available FFPE archival tumor tissues were collected for gene expression analysis. However, despite the small patient number, our findings concur with those of previous reports on other cancer types that demonstrated the significance of B cells in response to ICI treatment. Second, our patient cohort appears to have been heavily pretreated prior to receiving anti-PD-1 and anti-PD-L1 monotherapy or anti-PD-1/PD-L1-based immunotherapy combination and had worse clinical outcome than previous phase III studies. Third, we did not explore the association of TLS with CB. Because the gene list included in the NanoString platform is relatively limited, not all genes that have ever been reported to constitute the TLS signature were available. We thus did not explore the significance of TLS signature in the current study. Forth, the spatial profiling and the relative location or potential interaction of B cells with other immune cells were not investigated in this study (32). Lastly, this study merely provides an association between B cells in the TME and the clinical efficacy of anti-PD-1 treatment but no mechanistic insights.

## Conclusion

In summary, our study shows that B cells in the TME were associated with efficacy and improved outcomes in patients with ESCC undergoing anti-PD-1/PD-L1-based immunotherapy. This observation warrants additional confirmatory studies with larger patient cohorts.

## Data Availability Statement

The original contributions presented in the study are included in the article/**Supplementary Material**, further inquiries can be directed to the corresponding author.

## Ethics Statement

The studies involving human participants were reviewed and approved by National Taiwan University Hospital Research Ethical Committee B. The patients/participants provided their written informed consent to participate in this study.

## Author Contributions

J-CG: Conceptualization, Methodology, Investigation, Data Curation, Writing - Original Draft, Funding acquisition. C-LH: Methodology, Software, Validation, Formal analysis, Visualization. Y-LH: Investigation, Data Curation. C-CL: Investigation, Data Curation. T-CH: Investigation, Data Curation. I-CW: Investigation, Resources, Data Curation. C-YL: Investigation, Resources, Data Curation. M-YL: Investigation, Resources, Data Curation. H-YK: Investigation, Resources, Data Curation. A-LC: Conceptualization, Writing - Review & Editing. C-HH: Conceptualization, Investigation, Resources, Writing - Review & Editing, Supervision, Funding acquisition. All authors contributed to the article and approved the submitted version.

## Funding

This work was supported in part by the Ministry of Science and Technology, ROC (MOST 105-2314-B-002-186-MY3, MOST 106-2314-B-002-135-, MOST 107-2314-B-002-199-, MOST 108-2314-B-002-096-, MOST 108-2314-B-002-076-MY3, and MOST 109-2314-B-002-231-); Ministry of Health and Welfare, ROC (MOHW 107-TDU-B-211-114017 and MOHW 111-TDU-B-221-114006); National Taiwan University Hospital (NTUH 106-N3695); and National Taiwan University Cancer Center (NTUCCS-109-02, NTUCCS-110-10, and NTUCC-111-05). The funding agencies were not involved in the study design; collection, analysis, or interpretation of data or the writing of the manuscript.

## Conflict of Interest

C-HH reports grants from Ministry of Science and Technology, ROC, grants from Ministry of Health and Welfare, ROC, during the conduct of the study; grants and personal fees from Bristol-Myers Squibb, grants and personal fees from Ono Pharmaceutical, grants and personal fees from Merck Sharp & Dohme (MSD), grants and personal fees from Roche, personal fees from Merck Serono, grants from Beigene, grants from AstraZeneca, outside the submitted work. J-CG reports grants from Ministry of Science and Technology, ROC, grants from National Taiwan University Hospital, grants from National Taiwan University Cancer Center, during the conduct of the study; personal fees from Bristol-Myers Squibb, personal fees from Ono Pharmaceutical, personal fees from Merck Sharp & Dohme (MSD) outside the submitted work. I-CW, C-YL, and M-YL report personal fees from Bristol-Myers Squibb, personal fees from Ono Pharmaceutical, outside the submitted work.

The remaining authors declare that the research was conducted in the absence of any commercial or financial relationships that could be construed as a potential conflict of interest.

## Publisher’s Note

All claims expressed in this article are solely those of the authors and do not necessarily represent those of their affiliated organizations, or those of the publisher, the editors and the reviewers. Any product that may be evaluated in this article, or claim that may be made by its manufacturer, is not guaranteed or endorsed by the publisher.

## Supplementary Material

Supplementary Figure 1 Scatter plots of the B cell signature **(A, B)** and immunohistochemistry stromal CD19 **(C, D)** expression levels as indicated between CB and non-CB groups (CCRT, concurrent chemoradiotherapy; Tx, treatment).
