# Supplementary figures and images for: B Cells in Tumor Microenvironment Associated With The Clinical Benefit to Programmed Cell Death Protein-1 Blockade Therapy in Patients With Advanced Esophageal Squamous Cell Carcinoma
Source: Front Oncol. 2022 Jun 29;12:879398. doi: 10.3389/fonc.2022.879398 (PMC9276977; doi:10.3389/fonc.2022.879398)

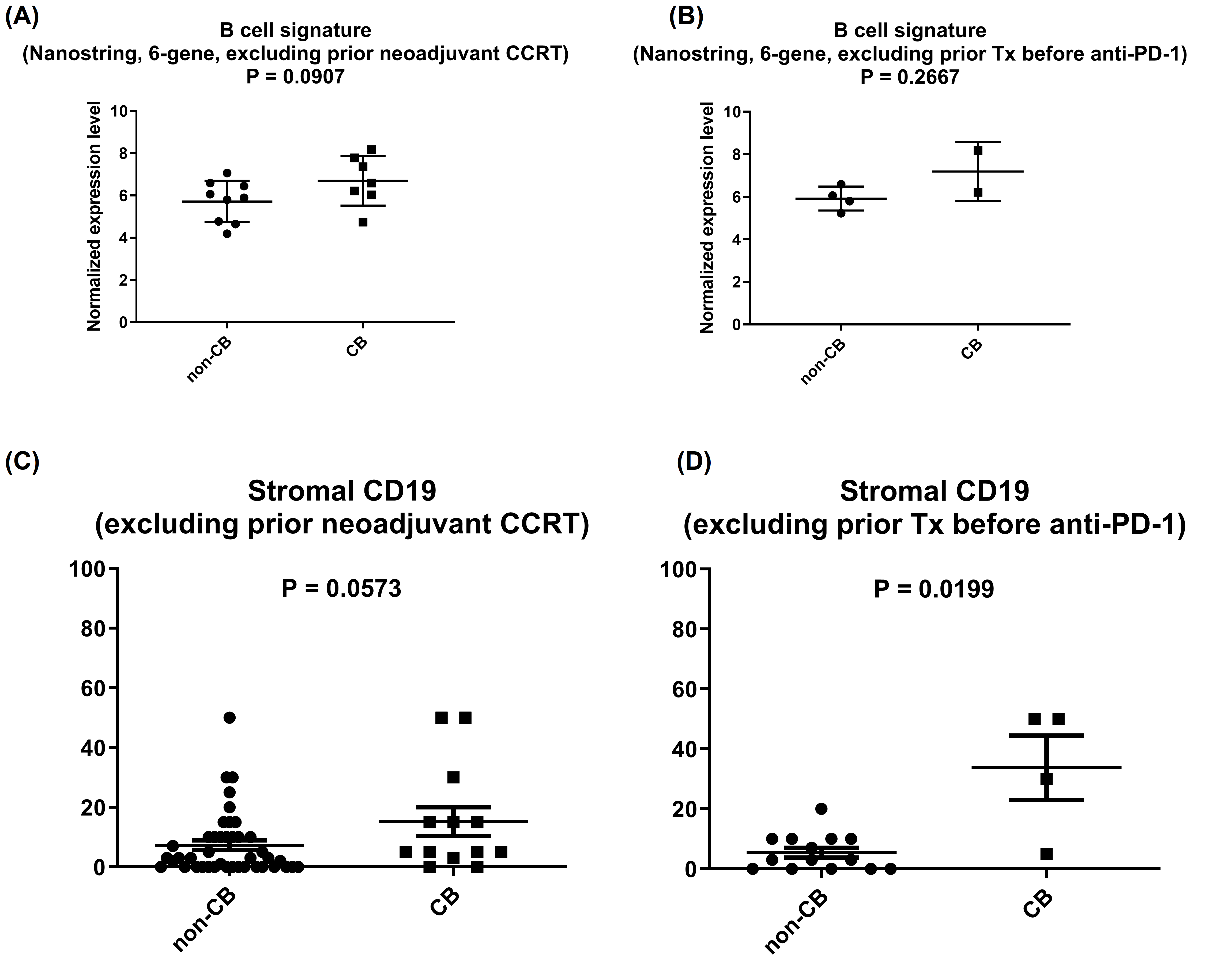

Supplement: Supplementary Figure 1 — Scatter plots of the B cell signature (A, B) and immunohistochemistry stromal CD19 (C, D) expression levels as indicated between CB and non-CB groups (CCRT, concurrent chemoradiotherapy; Tx, treatment). [file Image_1.tif]
