# Supplementary material for: B Cells in Tumor Microenvironment Associated With The Clinical Benefit to Programmed Cell Death Protein-1 Blockade Therapy in Patients With Advanced Esophageal Squamous Cell Carcinoma
Source: Front Oncol. 2022 Jun 29;12:879398. doi: 10.3389/fonc.2022.879398 (PMC9276977; doi:10.3389/fonc.2022.879398)
Supplement: Supplementary file 2 [file Table_1.docx]

**Supplement Table 1. Archival tissue source classified by site and prior neoadjuvant therapy.**

| **Gene expression analysis cohort (N=25)** | | | |
| --- | --- | --- | --- |
|  | Primary site with neoadjuvant therapy | Primary site without neoadjuvant therapy | Recurrence or metastasis site |
| Clinical benefit (CB) | 2 | 2 | 5 |
| Non-CB | 7 | 1 | 8 |
|  | | | |
| **Immunohistochemistry cohort (N=64)** | | | |
|  | Primary site with neoadjuvant therapy | Primary site without neoadjuvant therapy | Recurrence or metastasis site |
| CB | 3 | 7 | 6 |
| Non-CB | 6 | 23 | 19 |
